# Microscopic inspection and tracking of single upconversion nanoparticles in living cells

**DOI:** 10.1038/lsa.2018.7

**Published:** 2018-04-06

**Authors:** Fan Wang, Shihui Wen, Hao He, Baoming Wang, Zhiguang Zhou, Olga Shimoni, Dayong Jin

**Affiliations:** 1 Institute for Biomedical Materials and Devices (IBMD), Faculty of Science, University of Technology Sydney, Sydney, New South Wales 2007, Australia

**Nanoparticles have become new tools for cell biology imaging^1^, sub-cellular sensing^2^, super-resolution imaging^3, 4^ and drug delivery^5^. Long-term 3D tracking of nanoparticles and their intracellular motions have advanced the understanding of endocytosis and exocytosis as well as of active transport processes^6, 7, 8^. The sophisticated operation of correlative optical-electron microscopy^9, 10^ and scientific-grade cameras is often used to study intercellular processes. Nonetheless, most of these studies are still limited by the insufficient sensitivity for separating a single nanoparticle from a cluster of nanoparticles or their aggregates^8, 11, 12^. Here we report that our eyes can track a single fluorescent nanoparticle that emits over 4000 photons per 100 milliseconds under a simple microscope setup. By tracking a single nanoparticle with high temporal, spectral and spatial resolution, we show the measurement of the local viscosity of the intracellular environment. Moreover, beyond the colour domain and 3D position, we introduce excitation power density as the fifth dimension for our eyes to simultaneously discriminate multiple sets of single nanoparticles.**

By introducing thousands of photon sensitizers (i.e., Yb^3+^ ions) and activators ions (i.e., Tm^3+^ and Er^3+^ ions) to form an energy transfer network within a single nanoparticle, upconversion nanoparticles (UCNPs) can up-convert low-energy near-infrared (NIR) photons into high-energy visible emissions^13^. UCNPs emit tunable multi-colour emissions under single-wavelength excitation for multiplexed sensing with low cytotoxicity and high chemical/photostabilities for biomedical applications^14^. Non-bleaching and non-blinking UCNPs are among the best probes for long-term tracking studies^7^, autofluorescence-free biomolecular sensing^15^, super-resolution microscopy imaging^4^, *in vivo* bio-imaging^16^ and light-triggered nanomedicine applications^17^. UCNPs are the most efficient materials for ultra-low power multiphoton microscopy and deep-tissue imaging^18^.

Here we show a series of monodispersed UCNPs with a brightness that already meets the requirement for our eyes to observe single nanoparticles through a microscope. Figure 1a shows an upconversion fluorescence system built for the purpose of observing single nanoparticles. We performed a definitive vision test (Supplementary Table S1 and Figure 1b), where we individually tested 14 volunteers (overall, 28 eyes) to determine the number of emitted photons from single nanoparticle required to be distinguished by a human eye. We identified that at least 4186 photons per 100 ms are required for all tested eyes to see two separate blue nanoparticles (Figure 1b, region R1). In region R2, 17 eyes failed to recognize the blue colour, but the two particles were still distinguishable. In region R3, 21 eyes barely distinguished the spatially separated two nanoparticles, while region R4 has been identified as an insufficient number of photons to differentiate the two nanoparticles.

Figure 1c shows a series of different batches of UCNPs purposely synthesized to cover a large range of representative sizes and emission properties (see Supplementary Information Section 2 for the details of characterization). Remarkably, their emission is highly uniform, which provides the foundation for this work in distinguishing single UCNPs from their clusters, either from images recorded by a camera or through real-time observation by the eyes. Notably, single UCNPs can be detected under low excitation power densities. As shown in Figure 1b, there are 78 photons per 100 ms detected from the 4-photon upconversion emission band (455 nm) under an excitation power density as low as 320 W cm^−2^, and even the achieved intensity of 4186 photons per 100 ms for naked eye inspection requires a power density of approximately only 12 kW cm^−2^, which is almost four orders of magnitude smaller than the excitation power required in two-photon microscopy^19^.

Due to the optical diffraction limit, conventional far-field fluorescence microscopy does not have sufficient resolution to determine the number of nanoparticles when they are too close to each other. Approaches such as correlative electron microscopy^9^ or the recently reported MINFLUX method^20^ can be applied to improve the resolution. A high-level of uniformity in the UCNPs (Figure 1c) provides the ability for observers to identify a threshold intensity for single-UCNP detection. The emitters with a brightness higher than this threshold value will be identified as several nanoparticles within the diffraction limit region (e.g., labelled by orange dots in Figure 2a). The threshold value measured by the system (Figure 1a) enables automatic single-nanoparticle detection (Figure 2a, processed data) in real-time by computer processing of a wide-field fluorescence image (Supplementary Information Section 5). Note that the processed computer data compensate for the non-uniform excitation field and provide an accurate single identification accuracy (100% accuracy, Supplementary Fig. S6). Remarkably, due to the background-free detection with the NIR excitation, non-blinking and non-bleaching features of the UCNPs, the human eye can also identify this threshold and recognize single UCNPs during microscopic inspection (Figure 2a, eye vision).

The importance of the real-time observation of single cellular event comes from the detection of sub-cellular vesicles and protein movements and understanding their interactions in the complex cellular function. There are myriad of models^21^ that propose different sub-cellular functions, including cytoskeleton re-arrangement^22^, protein dynamics, organelle movement and cooperation^23, 24^, but until now, options for the real-time observation of these models in living cells have been limited. As Figure 2b shows, in the three typical 2D images taken at different heights, the high brightness of the photostable UCNPs is detectable not only in a dark room but also under bright-field illumination, providing the ability to identify the position of a single nanoparticle within a living cell and establishing a powerful tool for examining intercellular re-organization and trafficking.

Herein, by recording the data and offline analysis of nanoparticles inside living cells, the 3D trajectories of seven spots of single particles (# 2, 3, 4 and 6) and clusters (# 1, 5 and 7) within a single cell for an observation period of 21 s (Figure 2c) clearly show the heterogeneous dynamics of each single nanoparticle and cluster, highlighting the ability to precisely distinguish the transition between different dynamic phases of the transport of a single nanoparticle. The cumulative displacements (Figure 2d) and their corresponding mean-square displacement figures (Figure 2e) show that most of the particles exhibit ‘confined’ particle diffusion, which is most likely associated with nanoparticle movement inside the sub-cellular vesicle. Interestingly, particle #2 demonstrates a slightly higher motility as well as a higher diffusion coefficient of 0.18 μm^2^ s^−1^ (Figure 2f). Recently, Liu *et al.*^10^ established that the motility of gold nanoparticles in early endosomes is slightly higher than that in late endosomes/lysosomes. Our observation corresponds with those results well, as particles #1, 4, 5, 6 and 7 are all located in the perinuclear area that is similar to the location of late endosomes/lysosomes, while particle #2 is closer to the cell membrane (early endosome).

Remarkably, particle #3 moves relatively fast and exhibits two-phase particle movement, reaching a speed of 3 μm s^−1^ during phase II with a specific direction. Such a movement is associated with the active migration of molecular motor proteins on microtubules or actin filaments^8, 10, 25^. Figure 2f further illustrates that during phase II, particle #3 reaches a diffusion coefficient as large as 0.52±0.04 μm^2^ s^−1^, suggesting that its movement occurs within a transport channel. The much lower diffusion coefficients of the other particles and clusters are in good agreement with the values reported for the random diffusion of nanoparticles inside cells^7, 26^.

Our approach further enables the quantitative study of the localized environment viscosity, the knowledge of which provides powerful insight into protein dynamics, as the local viscosity contributes to the specific functioning of intracellular proteins^21^. Figure 2g shows that particles #4 and #6 reside inside late endosomes/lysosomes and exhibit low motility, which also corresponds to a higher viscosity inside those organelles (166±13 and 184±14 cP, respectively). The viscosity of early endosomes, where particle #2 is presumably located, is lower (41±4 cP)^27^, which is associated with more a dilute environment. In contrast to confined nanoparticles, particle #3 exhibits a high motility that corresponds to a lower viscosity (11.6±0.9 cP) in its vicinity (cytoplasm). The lower viscosity in the cytoplasm promotes higher hydration, which leads to higher protein functionality.

To further demonstrate the power of our approach in resolving single nanoparticles, we focused on tracking a diffraction-limit spot containing multiple UCNPs. Figure 3a–e show two UCNPs tightly confined within an ∼200 nm region for the first 168 s that are separated but within close proximity to each other. The intensity-based analysis (Figure 3d) clearly identifies one cluster (P1&P2) and two individual single nanoparticles (P1, P2). We further calculated Pearson’s correlation coefficient (see Supplementary Information Section 6) and obtained a value of 0.46 (*P*<0.05), which is considered to be a moderate positive correlation, indicating that the two UCNPs are not aggregated but have a degree of independency.

Figure 3f and g show that both the UCNPs are first retained in a confined area, possibly between actin filaments. After separation into singles nanoparticles (phase I), they proceed to move with a speed similar to that before separation. During phase II, both separated nanoparticles begin to move faster.

Interestingly, the diffusion coefficient analysis (Figure 3h) reveals that the diffusion coefficients for P1&P2, P1 in phase I (P1_I_) and P2 in phase I (P2_I_) have similar values, which also suggests that the two separate nanoparticles are confined in the same location; an aggregate with a larger size should have a smaller value. The viscosity calculation (Figure 3i) shows a very high viscosity value for P1 and P2, which most likely does not reflect the local viscosity but rather reflects the immobilized state of the nanoparticles associated with cell structural components, i.e., trapping between actin filaments.

The UCNPs presented in this work are not only bright but also display excitation-power-dependent properties, and Supplementary Figure S3 shows that high concentrations of Tm^3+^-doped UCNPs only turn on at relatively high excitation power densities^28^. This result offers a new dimension for the simultaneous imaging and tracking of multiple kinds of single nanoparticles. Figure 4 demonstrates the potential of this fifth untapped dimension independent of the conventional colour channels for optical multiplexed tracking of single nanoparticles in a 3D cellular environment, which is useful because it gives the ability of colour-blind observers to use upconversion fluorescence microscopes.

In summary, here, we have realized a library of UCNPs that allows the human eye to distinguish single nanoparticles in living cells through a microscope. This unique capability further enables the accurate measurement of localized intercellular environment viscosities for functional super-resolution imaging. Time-resolved imaging of lifetime-tunable UCNPs^29^ will enable multiplexed imaging and super-resolution imaging of sub-cellular structures. With both their excitation and emission bands in the NIR optically transparent biological window, the UCNPs demonstrated in this work make single nanoparticle tracking in deep tissue feasible.

## Figures and Tables

**Figure 1:**
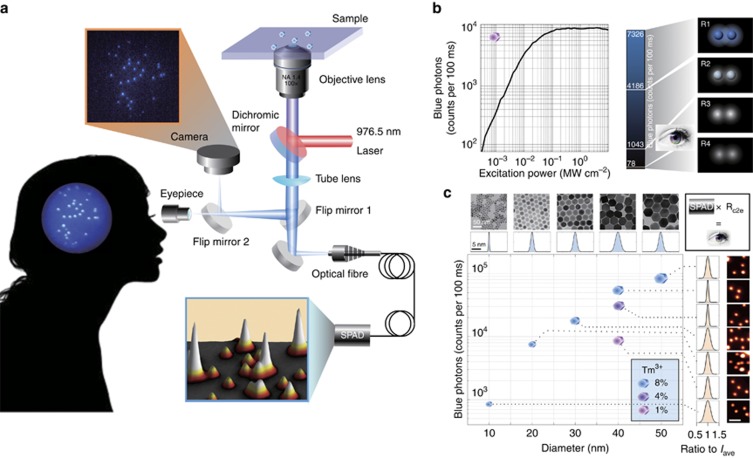
Schematic diagram and characterization results of a library of upconversion nanoparticles for testing human vision through a microscope. (**a**) Microscopy system that compares the sensitivity of a CCD camera and our eyes (switched by a flap mirror) in the observation of single UCNPs. The photons emitted from each single UCNP are collected by an optical fibre (1.01 Airy disk) and counted by a single photon avalanche detector (SPAD) for 100 ms, mimicking the duration time required for our brain to process images obtained from the same number of photons. (**b**) Excitation-power-dependent emission curve of 1 mol % Tm^3+^-doped UCNPs at the blue band (475±25 nm). We chose a region that contained two nearby UCNPs separated by 3 μm, which is equivalent to the 0.33 mm (Supplementary Equation S1) area of the central region of the human fovea (1.5 mm) where cone cells have their highest density (147000 cells per mm^2^). The sensitivity of our eyes is measured by the number of emission photons and categorized into four cognitive regions (R1: colour imaging; R2: mono-colour imaging; R3: inferior imaging; R4; blur imaging), which varied slightly among the 28 eyes of the 14 volunteers. (**c**) The size (transmission electron microscope images) and intensity (confocal scanning) at the blue band of a library of precisely controlled synthesized UCNPs, including peak sizes of 9.98, 22.25, 31.96, 39.78 and 50.21 nm and different Tm^3+^ doping concentrations of 1, 4 and 8 mol% for the ∼40 nm UCNPs. The standard deviation (s.d.) of the size varied from 6.23 to 17.4% from the average intensity (*I*_ave_). The photon counts used in this paper are the photon counts that reach a human cornea (*I*_eye_). It can be calculated according to Supplementary Equation S2 (*I*_eye_=*I*_con_/*R*_c2e_, Supplementary Information Section 3), where Icon is the photon counts measured by SPAD and *R*_c2e_ is the converting ratio.

**Figure 2:**
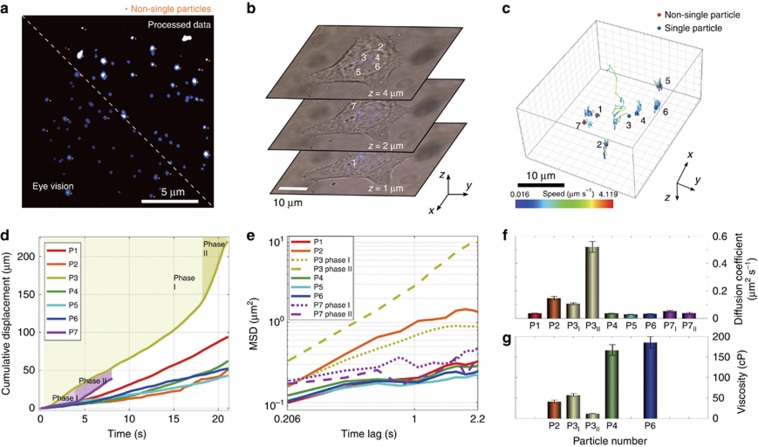
Wide-field upconversion fluorescence microscopy method for determining single nanoparticles for long-term 3D tracking and measurement of the local intracellular viscosity. (**a**) The bottom left image was recorded by a CCD camera (Nikon DS-2Mv) and adjusted (exposure time 150 ms; gain at 4) to show the equivalent intensity level of the image observed by the eye. The top right image was recorded by the same CCD image, and the intensity of each nanoparticle spot was compensated by considering the Gaussian excitation pattern and the power-dependent emission property of the UCNPs (doped with 1 mol% Tm^3+^) (see Supplementary Information Section 5). Both half images allow for the determination of single UCNPs and clusters or spots containing more than one UCNP, marked by an orange dot. (**b**) Three representative single-cell images containing seven UCNP spots (doped with 4 mol% Tm^3+^) at different heights of 1, 2 and 4 μm recorded by a CCD camera in video mode (4.75 frames per second; the exposure time of each frame is 150 ms; gain at 4) during the *z*-axis scanning process. The high brightness of each single UCNP allows for the real-time recording of the upconversion fluorescence images under bright-field illumination. (**c**) 3D trajectories of seven numbered upconversion spots, including four single UCNPs (#2, 3, 4 and 6), marked by blue dots and three non-single UCNPs (#1, 5 and 7), marked by orange dots, observed for 21 s by a 368.85 s tracked video (Supplementary Movie S1). (**d**) Cumulative displacement and (**e**) mean-square displacement (MSD) analysis of the seven spots showing their movement speeds at different times; particles #3 and #7 show two phases of movement. The MSDs are plotted as a function of time lag, which is the time required for the particle to be displaced through diffusion (see Supplementary Information Section 6). (**f**) Fitting results of the diffusion coefficients for the seven UCNP spots, containing two phases of movement for particles #3 and #7. (**g**) Localized viscosity measurements by the four single UCNPs, including the two movement environments of single particle #3.

**Figure 3:**
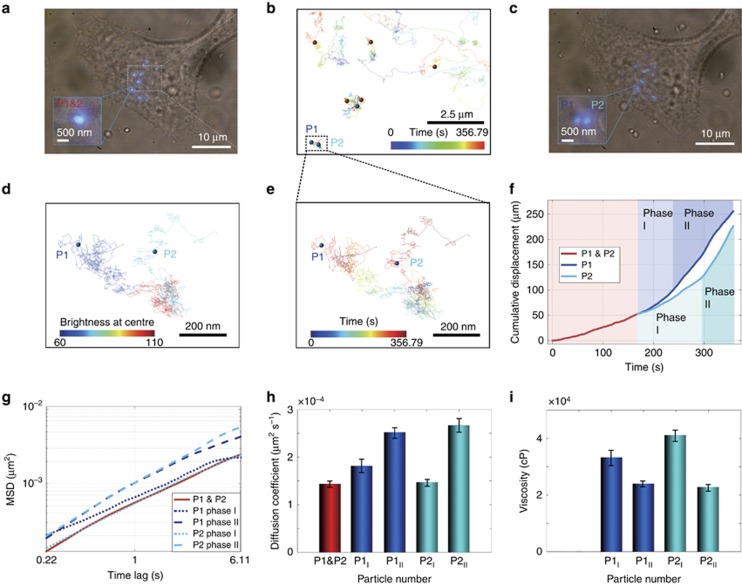
High-resolution long-term tracking of two single UCNPs confined within a diffraction-limit spot and their escape process. (**a**) Bright-field and epifluorescence image of a single cell taken at 0.21 s from Supplementary Video S2. The insert image is the cluster of interest in this study, which contains two single 40 nm 4 mol% Tm^3+^-doped UCNPs. (**b**) 2D tracking of the 8.5 × 6.4 μm area for the period from 10.5 s to 367.29 s. (**c**) Bright-field and epifluorescence image of a single cell at 372.54 s from Supplementary Video S2. (**d**) Intensity map of the two nanoparticles showing the transformation from a diffraction-limit cluster (red) to two independent nanoparticles (blue for particle 1 and light blue for particle 2). (**e**) 2D pathways of the two tracked single nanoparticles from their confinement to their escape from their local environment over time (colour coded). (**f**) Cumulative displacement and (**g**) MSD analysis of the cluster and its separation into two single nanoparticles, each of which later experienced two phases of movement at different times. (**h**) Calculated diffusion coefficient and (**i**) resultant local viscosity measurements for the two separated single UCNPs.

**Figure 4:**
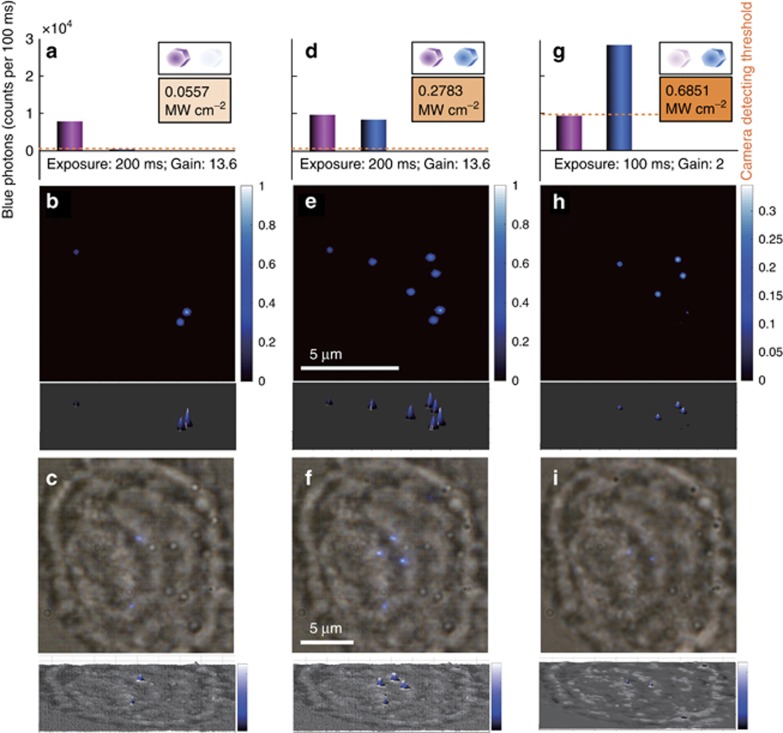
Multiplexed imaging strategy for tracking multiple types of UCNPs at the same emission colour band. (**a**–**c**) Under an average excitation power density of 0.0557 MW cm^−2^ and with the CCD camera set for a 200 ms exposure time at a gain value of 13.6, only the blue emission of the 1 mol% Tm^3+^-doped UCNPs are sufficiently bright for single UCNP observation. (**d**–**f**) Under a medium excitation power density of 0.2783 MW cm^−2^ and with the same settings of the CCD camera, the 8 mol% Tm^3+^ UCNPs start to emit a comparable amount of upconversion luminescence to that of the 1 mol% Tm^3+^ UCNPs. (**g**–**i**) Under a relatively high excitation intensity, e.g., 0.6851 MW cm^−2^ and above, the 8 mol% Tm^3+^ UCNPs become so bright that they saturate the camera; thus, a reduced gain with a value of 2 and an exposure time of 100 ms are applied for the image recording. Under this condition, the signals from each single 1 mol% Tm^3+^ UCNP become lower than the detection threshold of the camera (orange dotted lines), making it undetectable compared with the brightly emitting 8 mol% UCNPs. (**b**, **e**, **h**) are the background-free upconversion images. (**c**, **f**, **i**) are the upconversion images in a living cell when bright-field illumination is on. To compare the recorded emission intensity of each UCNP, each upconversion image is also presented as a 3D intensity plot below each image.

## References

[bib1] Rees P, Wills JW, Brown MR, Tonkin J, Holton MD et al. Nanoparticle vesicle encoding for imaging and tracking cell populations. Nat Methods 2014; 11: 1177–1181. 25218182 10.1038/nmeth.3105

[bib2] Brites CDS, Fuertes MC, Angelomé PC, Martínez ED, Lima PP et al. Tethering luminescent thermometry and plasmonics: light manipulation to assess real-time thermal flow in nanoarchitectures. Nano Lett 2017; 17: 4746–4752. 28686837 10.1021/acs.nanolett.7b01433

[bib3] Barbiero M, Castelletto S, Gan XS, Gu M. Spin-manipulated nanoscopy for single nitrogen-vacancy center localizations in nanodiamonds. Light Sci Appl 2017; 6: e17085. 10.1038/lsa.2017.85 PMC6062043 30167213

[bib4] Liu YJ, Lu YQ, Yang XS, Zheng XL, Wen SH et al. Amplified stimulated emission in upconversion nanoparticles for super-resolution nanoscopy. Nature 2017; 543: 229–233. 28225761 10.1038/nature21366

[bib5] Hinde E, Thammasiraphop K, Duong HTT, Yeow J, Karagoz B et al. Pair correlation microscopy reveals the role of nanoparticle shape in intracellular transport and site of drug release. Nat Nanotechnol 2017; 12: 81–89. 27618255 10.1038/nnano.2016.160

[bib6] Bhatia D, Arumugam S, Nasilowski M, Joshi H, Wunder C et al. Quantum dot-loaded monofunctionalized DNA icosahedra for single-particle tracking of endocytic pathways. Nat Nanotechnol 2016; 11: 1112–1119. 27548358 10.1038/nnano.2016.150 PMC5122452

[bib7] Nam SH, Bae YM, Park YI, Kim JH, Kim HM et al. Long-term real-time tracking of lanthanide ion doped upconverting nanoparticles in living cells. Angew Chem Int Ed 2011; 50: 6093–6097. 10.1002/anie.201007979 21574220

[bib8] Jo HL, Song YH, Park J, Jo EJ, Goh Y et al. Fast and background-free three-dimensional (3D) live-cell imaging with lanthanide-doped upconverting nanoparticles. Nanoscale 2015; 7: 19397–19402. 26537159 10.1039/c5nr05875a

[bib9] Albanese A, Chan WCW. Effect of gold nanoparticle aggregation on cell uptake and toxicity. ACS Nano 2011; 5: 5478–5489. 21692495 10.1021/nn2007496

[bib10] Liu MM, Li Q, Liang L, Li J, Wang K et al. Real-time visualization of clustering and intracellular transport of gold nanoparticles by correlative imaging. Nat Commun 2017; 8: 15646. 28561031 10.1038/ncomms15646 PMC5460036

[bib11] Lowe AR, Siegel JJ, Kalab P, Siu M, Weis K et al. Selectivity mechanism of the nuclear pore complex characterized by single cargo tracking. Nature 2010; 467: 600–603. 20811366 10.1038/nature09285 PMC2948059

[bib12] Fu CC, Lee HY, Chen K, Lim TS, Wu HY et al. Characterization and application of single fluorescent nanodiamonds as cellular biomarkers. Proc Natl Acad Sci USA 2007; 104: 727–732. 17213326 10.1073/pnas.0605409104 PMC1783382

[bib13] Zhou B, Shi BY, Jin DY, Liu XG. Controlling upconversion nanocrystals for emerging applications. Nat Nanotechnol 2015; 10: 924–936. 26530022 10.1038/nnano.2015.251

[bib14] Yang YM, Velmurugan B, Liu XG, Xing BG. NIR photoresponsive crosslinked upconverting nanocarriers toward selective intracellular drug release. Small 2013; 9: 2937–2944. 23554151 10.1002/smll.201201765

[bib15] van de Rijke F, Zijlmans H, Li S, Vail T, Raap AK et al. Up-converting phosphor reporters for nucleic acid microarrays. Nat Biotechnol 2001; 19: 273–276. 11231563 10.1038/85734

[bib16] Nyk M, Kumar R, Ohulchanskyy TY, Bergey EJ, Prasad PN. High contrast *in vitro* and *in vivo* photoluminescence bioimaging using near infrared to near infrared up-conversion in Tm^3+^ and Yb^3+^ doped fluoride nanophosphors. Nano Lett 2008; 8: 3834–3838. 18928324 10.1021/nl802223f PMC3523349

[bib17] Idris NM, Gnanasammandhan MK, Zhang J, Ho PC, Mahendran R et al. *In vivo* photodynamic therapy using upconversion nanoparticles as remote-controlled nanotransducers. Nat Med 2012; 18: 1580–1585. 22983397 10.1038/nm.2933

[bib18] Watson JM, Marion SL, Rice PF, Utzinger U, Brewer MA et al. Two-photon excited fluorescence imaging of endogenous contrast in a mouse model of ovarian cancer. Lasers Surg Med 2013; 45: 155–166. 23362124 10.1002/lsm.22115 PMC4566968

[bib19] Moerner WE, Fromm DP. Methods of single-molecule fluorescence spectroscopy and microscopy. Rev Sci Instrum 2003; 74: 3597–3619.

[bib20] Balzarotti F, Eilers Y, Gwosch KC, Gynnå AH, Westphal V et al. Nanometer resolution imaging and tracking of fluorescent molecules with minimal photon fluxes. Science 2017; 355: 606–612. 28008086 10.1126/science.aak9913

[bib21] Frauenfelder H, Chen G, Berendzen J, Fenimore PW, Jansson H et al. A unified model of protein dynamics. Proc Natl Acad Sci USA 2009; 106: 5129–5134. 19251640 10.1073/pnas.0900336106 PMC2649210

[bib22] Fakhri N, Wessel AD, Willms C, Pasquali M, Klopfenstein DR et al. High-resolution mapping of intracellular fluctuations using carbon nanotubes. Science 2014; 344: 1031–1035. 24876498 10.1126/science.1250170

[bib23] Chu BB, Liao YC, Qi W, Xie C, Du XM et al. Cholesterol transport through lysosome-peroxisome membrane contacts. Cell 2015; 161: 291–306. 25860611 10.1016/j.cell.2015.02.019

[bib24] Valm AM, Cohen S, Legant WR, Melunis J, Hershberg U et al. Applying systems-level spectral imaging and analysis to reveal the organelle interactome. Nature 2017; 546: 162–167. 28538724 10.1038/nature22369 PMC5536967

[bib25] Schütz GJ, Schindler H, Schmidt T. Single-molecule microscopy on model membranes reveals anomalous diffusion. Biophys J 1997; 73: 1073–1080. 9251823 10.1016/S0006-3495(97)78139-6 PMC1181003

[bib26] Holtzer L, Meckel T, Schmidt T. Nanometric three-dimensional tracking of individual quantum dots in cells. Appl Phys Lett 2007; 90: 053902.

[bib27] Kuimova MK, Botchway SW, Parker AW, Balaz M, Collins HA et al. Imaging intracellular viscosity of a single cell during photoinduced cell death. Nat Chem 2009; 1: 69–73. 21378803 10.1038/nchem.120

[bib28] Zhao JB, Jin DY, Schartner EP, Lu YQ, Liu YJ et al. Single-nanocrystal sensitivity achieved by enhanced upconversion luminescence. Nat Nanotechnol 2013; 8: 729–734. 23995455 10.1038/nnano.2013.171

[bib29] Lu YQ, Zhao JB, Zhang R, Liu YJ, Liu DM et al. Tunable lifetime multiplexing using luminescent nanocrystals. Nat Photon 2014; 8: 32–36.

