# Supplementary material for: Microscopic inspection and tracking of single upconversion nanoparticles in living cells
Source: Light Sci Appl. 2018 Apr 6;7:18007–. doi: 10.1038/lsa.2018.7 (PMC5987356; doi:10.1038/lsa.2018.7)
Supplement: Supplementary Information [file lsa20187x1.docx]

**Supplementary Information**

**Microscopic inspection and tracking of single upconversion nanoparticles in living cells**

Fan Wang^1^*, Shihui Wen^1^, Hao He^1^, Baoming Wang^1^, Zhiguang Zhou^1^, Olga Shimoni^1^, Dayong Jin^1^*

^1^Institute for Biomedical Materials and Devices (IBMD), Faculty of Science, University of Technology Sydney, NSW 2007, Australia (*Correspondence and requests for materials should be addressed to [fan.wang@uts.edu.au](mailto:fan.wang@uts.edu.au) or [dayong.jin@uts.edu.au](mailto:dayong.jin@uts.edu.au))

# **Section 1: Synthesis of upconversion nanoparticles (UCNPs)**

**1.1 Synthesis of NaYF_4_: Yb, Tm nanocrystals**

NaYF_4_: Yb^3+^, Tm^3+^ nanocrystals with different Tm doping (from 0.5% to 8%) were synthesized according to our previously reported method^1, 2^. In a typical experiment, 1 mmol RECl_3_·6H_2_O (RE = Y, Yb, Tm) with the desired molar ratio were added to a flask containing 6 mL OA and 15 mL ODE. The mixture was heated to 160 °C under argon flow for 30 min to obtain a clear solution and then cooled down to about 50 °C, followed by the addition of 5 mL methanol solution of NH_4_F (4 mmol) and NaOH (2.5 mmol). After stirring for 30 min, the solution was heated to 80 °C under argon flow for 20 min to expel methanol, and then the solution was further heated to 310 °C for another 90 min. Finally, the reaction solution was cooled down to room temperature. The products were precipitated by ethanol and centrifuged (9000 rpm for 5 min), then washed 3 times with cyclohexane, ethanol and methanol to get the nanoparticles.

To get the nanoparticles with desired size or core-shell structure, layer-by-layer epitaxial growth has been employed. The shell precursors preparation is similar with that for the core nanoparticles synthesis, until the step where the reaction solution was slowly heated to 150°C and kept for 20 min. Instead of further heating to 300 °C to trigger nanocrystal growth, the solution was cooled down to room temperature to yield the shell precursors. For epitaxial growth, 0.15 mmol as-prepared core nanocrystals were added to a containing 6 ml OA and 6 ml ODE. The mixture was heated to 170 °C under argon for 30 min, and then further heated to 300 °C. Next, 0.25 ml as prepared shell precursors were injected into the reaction mixture and ripened at 300 °C for 4 min, followed by the same injection and ripening cycles for several times to get the nanocrystals with the desired size. Finally, the slurry was cooled down to room temperature and the formed nanocrystals were purified according to the same procedure used for the core nanocrystals.

The protocol for synthesizing 10 nm NaYF_4_:20%Yb^3+^,8%Tm^3+^ nanocrystals is similar to the one above for producing the core nanocrystals, except for the different amount of Na source. In a typical experiment, 0.4 mmol RECl_3_ (RE = Y, Yb, Tm) with the molar ratio of 72:20:8 were added to a 50 mL flask containing 6 mL OA and 6 mL ODE. The mixture was heated to 160 ℃ under argon for 30 min to obtain a clear solution and then cooled down to about 70 ℃, followed by the addition of 1.00 g of NaOA and 0.17 g NH_4_F. After stirring for 30 min, the solution was heated to 150 ℃ under argon for 20 min, and then the solution was further heated to 300 ℃ for another 45 min. Finally, the reaction solution was cooled down to room temperature, and nanoparticles were purified according to the same procedure used for the core nanocrystals.

**1.2 Preparation of hydrophilic UCNPs**

To convert OA caped UCNPs to hydrophilic, surface modification of UCNPs has been carried out via ligand exchange with a block copolymer composed of hydrophilic block poly (ethylene glycol) methyl ether acrylate and polymer block containing phosphate (POEGA-b-PMAEP). Phosphate groups on the block copolymer utilized as anchoring groups onto the surface of UCNPs due to high affinity.

In the typical procedure, 500 uL of UCNPs (20 mg mL^-1^) in cyclohexane were precipitated with ethanol. After centrifuge and discarding the solution, UCNPs were redispersed in 1 mL THF with assist of vortexing and sonication. Further, UCNPs were mixed with 1 mL of 5 mg mL^-1^ of POEGA-b-PMAEP in THF. The reaction was kept gentle shaking overnight at room temperature. Subsequently, 3 mL of water was added and shaked, following by addition of 1 mL of hexane to remove the OA. Once the oil phase has been discarded, the UCNPs dispersion has been kept in vacuo overnight to evaporate ret of organic solvents. Finally, the dispersion was dialyzed in 1 L of water for 24 h to remove the excess of POEGA-b-PMAEP polymer. The final concentration of hydrophilic UCNPs in water is 5 mg mL^-1^.

# **Section 2: UCNPs characterization**

**2.1 TEM size characterization**

The morphology of the formed materials was characterized via transmission electron microscopy (TEM) imaging (Philips CM10 TEM with Olympus Sis Megaview G2 Digital Camera) with an operating voltage of 100 kV. The samples were prepared by placing a drop of a dilute suspension of nanocrystals onto copper grids.


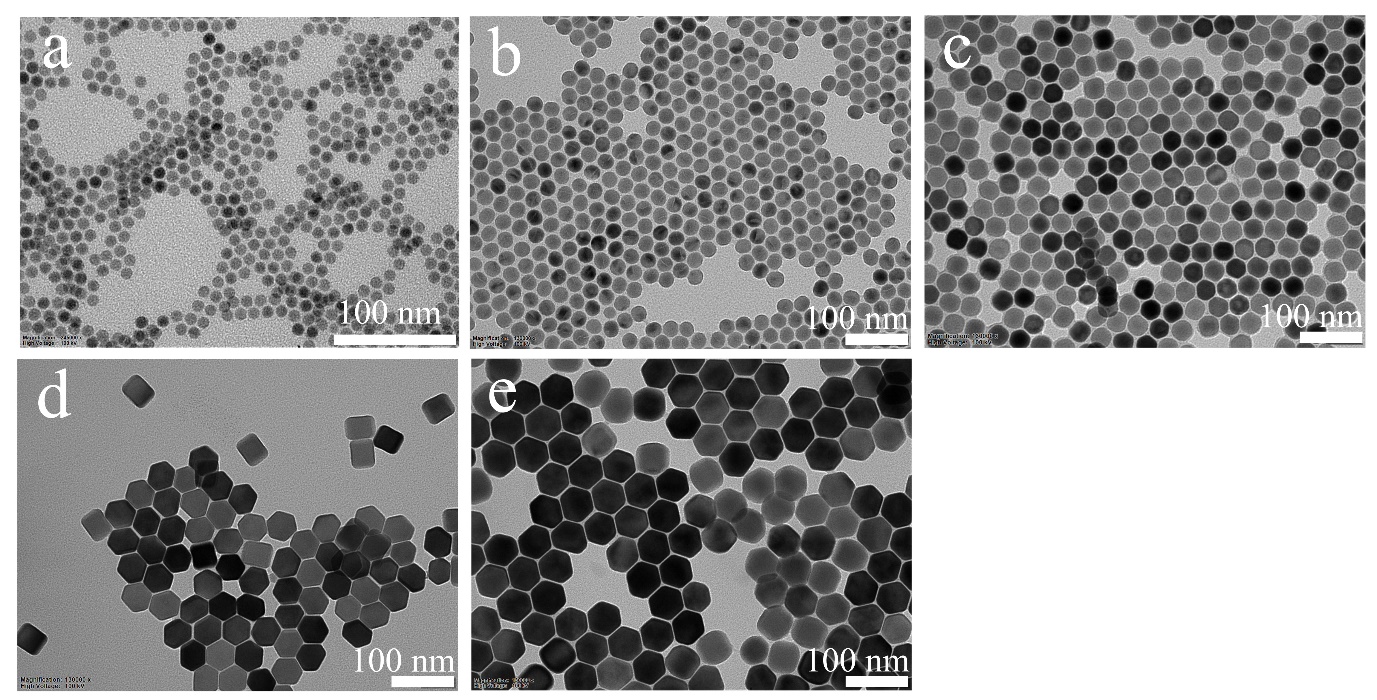


**Figure S1.** Typical TEM micrographs of 8% mol Tm^3+^ doped UCNPs with peak sizes of (a) 9.98 ± 0.39 nm, (b) 22.25 ± 1.05 nm, (c) 31.96 ± 1.33 nm, (d) 39.78 ± 1.93 nm, and (e) 50.21 ± 1.50 nm. TEM images in the main text Figure 1c were derived from these micrographs.

**2.2 Emission brightness**

Characterization of single UCNPs has been performed by a purpose-build scanning confocal system^3^, which is shown in Figure S2. A 976.5 nm laser is used to excite the UCNPs, with a home build power control unit including a half wave plate and a polarizer. The emission of UCNPs is collected by a high NA objective lens (Olympus, NA = 1.4), then focused by a tube lens to an optical fibre (working as confocal aperture Airy disk = 1.01), which is linked to either a single photon avalanche detector (SPAD). The scanning is achieved by x-y movement of the stage. The blue band emission collection is achieved by switching the filter wheel to a 475 ± 25 nm bandpass filter.

**
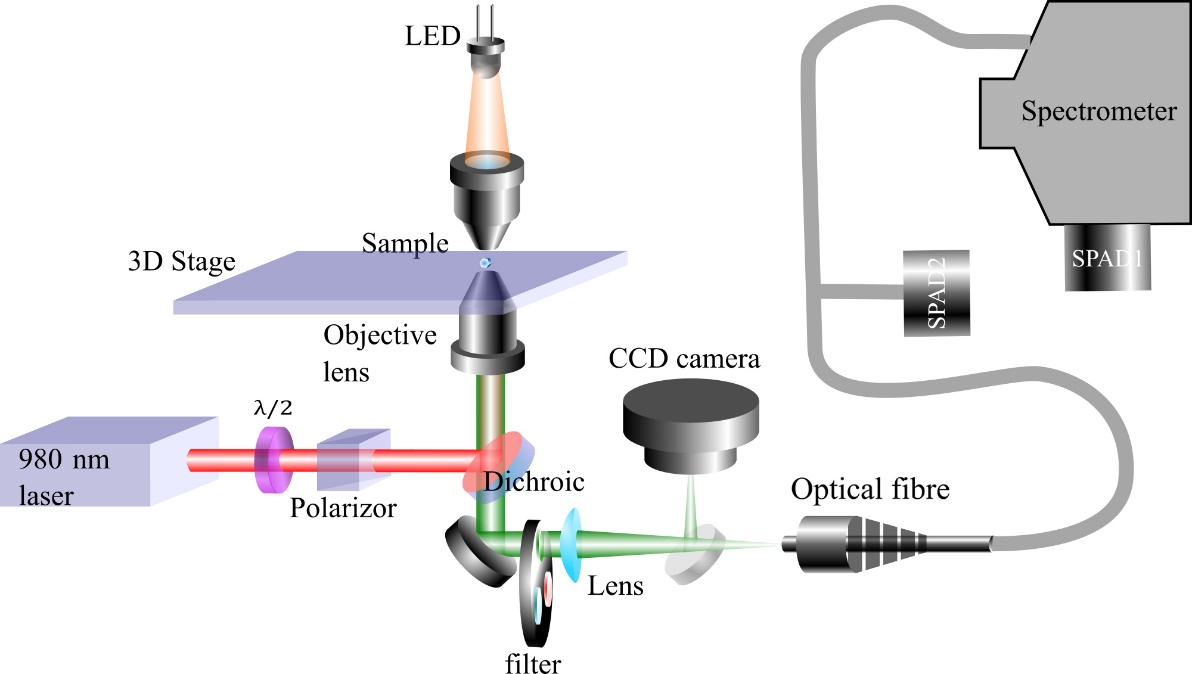
**

**Figure S2.** Scanning confocal system for single UCNP emission characterization.

Power dependent emission of UCNPs:


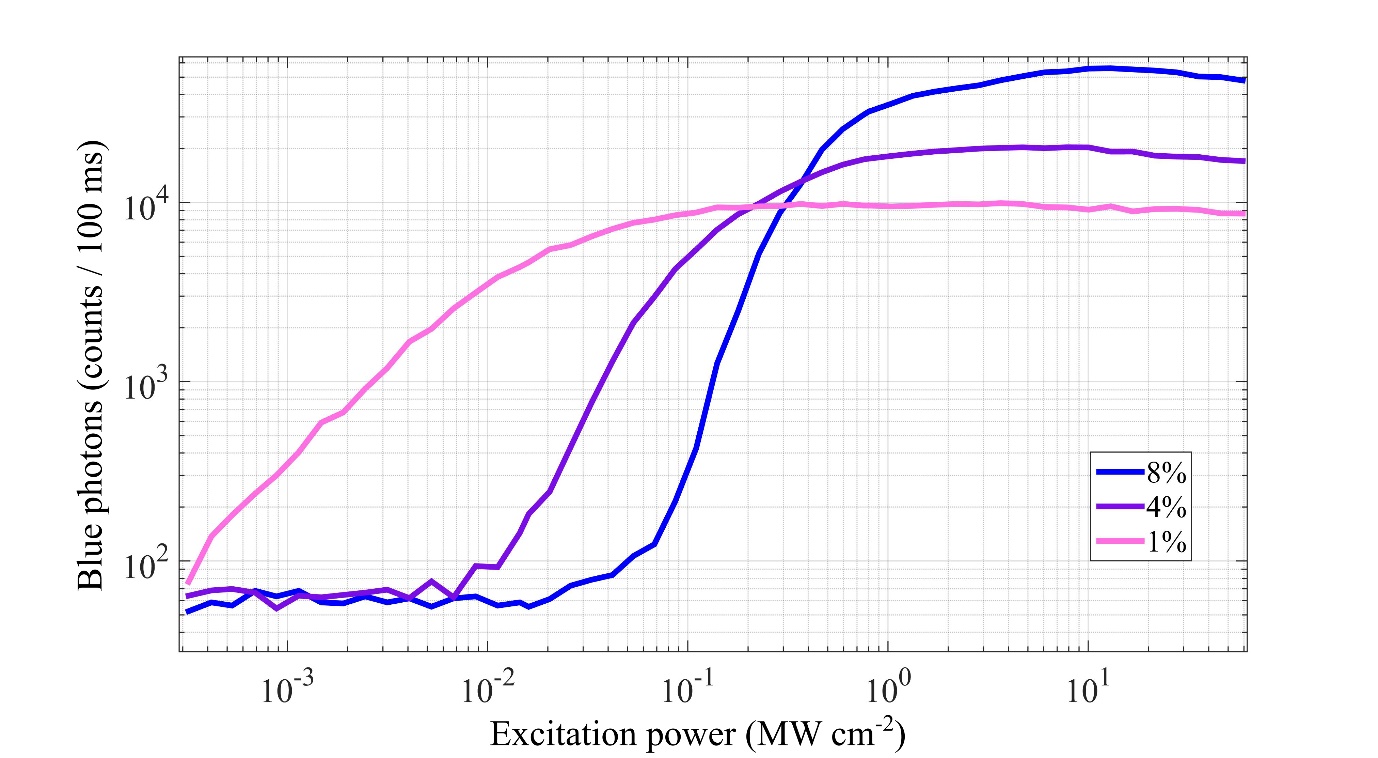


**Figure S3**. Blue band (475 ± 25 nm) power dependent emission response curve for single UCNP with Tm^3+^ doping concentration 8%, 4% and 1% mol. Here 8% and 1% mol Tm^3+^ doped UCNPs can be used to provide the fifth-dimension tracking of single UCNP as shown in Figure 4, since they have distinct power dependent response.

# **Section 3: Human vision test**

**3.1 Excitation beam**

Large area excitation is achieved by a 4f defocusing system as shown in Figure S4. For normal 4f system, the laser is focused on the focal plane of objective lens as shown in Figure S4a. By insetting a thin lens (defocusing lens) at the phase conjugate plane of back aperture of the objective lens, laser’s focusing position will be shifted as shown in Figure S4b, which result in an expended Gaussian beam on the focal plane of objective lens. Different focal length of defocusing lens will result in different spot size on the focal plane. For 200 mm, 150 mm and 100 mm focal lengths of defocusing lens, the Gaussian beam spot’s radiuses are 5.2 µm, 5.72 µm and 6.9 µm respectively. The excitation intensity at the centre of the beam can be estimated from UCNP’s emission counts under defocused beam excitation.


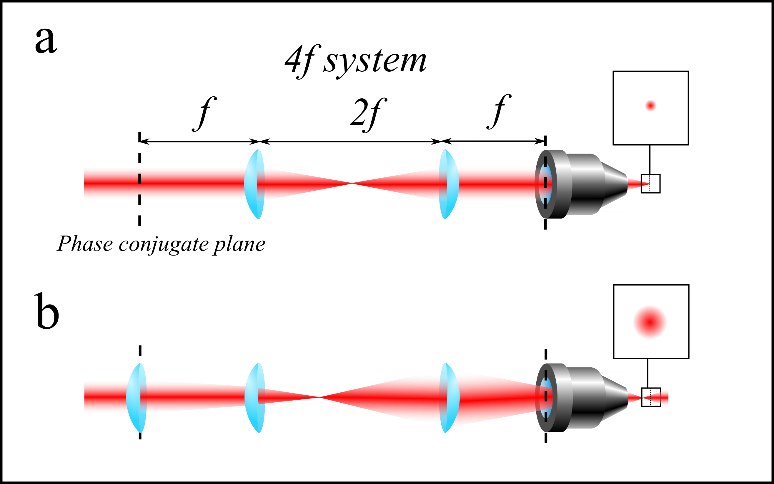


**Figure S4.** The schematic diagram of large area excitation. (a) Illustration of 4f system that is used in excitation beam path. (b) By insetting a defocusing lens at the phase conjugate plane of back aperture the, the input Gaussian beam can be defocused to a larger excitation spot. By changing the defocusing lens, the size of large excitation spot can be controlled.

**3.2 Optical systems**

The optical system for a human vision testing is shown in Figure 1a and Figure S2, where the CCD camera (Nikon DS-2Mv) and human eye are sharing the same wide-field epi-illumination image that can be switched by a flip mirror. The confocal photon counts are collected by SPAD. It is important to mention that the photon counts shown in Figure 1 and Figure 4 are the photon counts reach human cornea which is transferred from SPAD detected number as shown in section of eye photon value calculation.

To estimate the imaging area of vision system in human retina, we calculated the relationship between the image size in real plane and the image size in human retina as below:

$y_{fovea}=y_{real} \times M \times\frac{f_{eye}}{f_{eyepiece}}$ (S1)

Here *y_fovea_* is the image size on retina, *y_real_* is the image size in real plane, *M* is the microscope magnification number (*M* = 125 for our system), *f_eye_* is the focal length of human eye which is 22.3 mm^4^, *f_eyepiece_* is the focal length of eyepiece which is 25 mm. Therefore, 3 µm distance in real image plane is 0.335 mm in central of retina (fovea) through microscope system.

**3.3 Eye photon value calculation**

In this paper, cognition test of human vision is characterized by photon numbers picked up by eye (*I_eye_*), which is calculated based on the photon numbers collected by confocal microscopy (*I_con_*) using the formula:

*I_eye_=I_con_ / R_c2e_*  (S2)

*R_c2e_ = R_aperture_ *R_detector_*  (S3)

Here *R_c2e_* is the ratio to convert photon on detector of confocal system to photons on human cornea for vision testing system. *R_aperture_* is the ratio of photon number collected through a confocal aperture to the photon number collected without aperture. For our system, we have a 1.01 Airy disk for 455 nm. The measured *R_aperture_* is 0.6. The *R_detector_* is the detection efficiency includes detector’s reflection and coupling efficiency. In our system, *R_detector_* is 0.6828. Therefore, the *R_c2e_* is calculated as 2.442, which indicates that 1000 photons detected by SPAD detector is equivalent to 2442 photons on human cornea.

**3.4 Human vision test result**

| Volunteer No. | Gender | Glasses | Colour threshold photon L (100 ms) | Image threshold photon L (100 ms) | Colour threshold photon R (100 ms) | Image threshold photon R (100 ms) |
| --- | --- | --- | --- | --- | --- | --- |
| 1 | M | Y | 1043 | 789 | 335 | 335 |
| 2 | F | Y | 611 | 78 | 2799 | 161 |
| 3 | M | Y | 1043 | 161 | 1043 | 78 |
| 4 | F | N | 3687 | 1043 | 3687 | 335 |
| 5 | F | Y | 1600 | 335 | 3687 | 611 |
| 6 | F | Y | 1600 | 611 | 1043 | 611 |
| 7 | M | N | 1600 | 161 | 789 | 161 |
| 8 | F | N | 2799 | 611 | 2799 | 1043 |
| 9 | F | Y | 2799 | 161 | 611 | 78 |
| 10 | M | Y | 335 | 161 | 1043 | 611 |
| 11 | M | Y | 335 | 78 | 335 | 78 |
| 12 | M | N | 78 | 78 | 18 | 78 |
| 13 | M | Y | 2799 | 611 | 1600 | 611 |
| 14 | M | Y | 4186 | 335 | 4186 | 1043 |

**Table S1.** The Human vision imaging test results. Here M stands for Male, F is female. Y means the volunteer is wearing glasses, N is without glasses. Colour threshold photon is the number from which emission range volunteer cannot recognize colour. Image threshold photon is the number from which emission range volunteer cannot distinguish two single UCNPs which are separated by 3 µm. L stands left eye, R stands right eye. All the numbers are the photon values on human corner converted from SPAD measured value according to Equation S1.

# **Section 4: Cell culture and particle treatment**

**4.1 Cell culture**

A549 (American Type Culture Collection; mycoplasma free) cells were cultured in tissue culture flasks (25 cm^2^, Falcone) in humid atmosphere at 37 °C and 5% CO_2_. The cells were grown in GlutaMAX^TM^ RPMI1640 medium (Gibco), supplemented with 10% FBS (Gibco). Cells were subcultured when approximately 80% confluency has been reached using 0.25% trypsin-EDTA (Gibco) for cell detachment. One day before a particle tracking experiment, 10^4^ A549 cells in 2 mL cell medium were plated into a microscopy dish (35 mm glass-bottom dish, Fluorodish) and incubated overnight (37 °C, 5% CO^2^) until the cells became adherent again.

**4.2 Particle treatment**

Once the present media was removed, UCNPs dispersion (50 ng mL^-1^ dispersed in 500 μL cell medium) was added into the microscopy dish and the cells were incubated for 2 h (37 °C, 5% CO^2^). Finally, the media with UCNPs was removed, the cells were washed three times with Phosphate-buffered saline buffer (PBS, Gibco; PBS contains 1.5 mM KH_2_PO_4_, 155.2 mM NaCl, 2.7 mM Na_2_HPO_4_ and the pH is 7.2) and covered with 500 μL of cell media.

# **Section 5: Single UCNP selection method**

**5.1 Gaussian excitation pattern compensation**

Wide-field illumination by a coherent laser beam usually suffers from non-uniform excitation intensity across the field with a typical Gaussian distribution pattern, which influences our intensity-based approach to judging a single nanoparticle. To compensate such an artefact, we developed an excitation pattern correction approach to correct emission images that is based on the position of nanoparticles in the non-uniform Gaussian excitation field and the power-dependency curves of UCNPs.

The process of single UCNP determination takes into account both excitation beam profile and UCNP’s power dependent emission curve. The emission pattern *(E_emi_*) which indicates emission intensity of a single UCNP will vary in different positions of excitation beam. *E_emi_* can be calculated through *E_emi_ = PD* E_exc_*, where *PD* is the excitation power dependency of emission intensity of single UCNP and can be measured through single particle characterization. *E_exc_* is the calculated excitation beam profile with measured spot size. To compensate the excitation intensity reduction due to decreased excitation intensity away from centre of Gaussian beam spot, Figure S5b show a 4% mol Tm^3+^ doped UCNP pattern (*I_in_*). The compensation process is achieved through formula *I_out_ = I_in_ / E_emi_*. After this intensity compensation process, single UCNP can be determined by their emission intensity. For example, single 4% mol Tm^3+^ doped UCNP should give emission value smaller than 0.824. If the intensity is higher than 0.824 than the bright spot is considerate as a non-single nanoparticle. It is worth to mention that this method can be made into a Matlab plugin code to incorporate for the Imaris software that can automatically determine single UCNP during tracking process.

For human eyes, our brain can be well trained to compensate differences when observing intensity alterations across a non-uniform excitation field, and a reference single UCNP as an alternative approach can facilitate this judgement


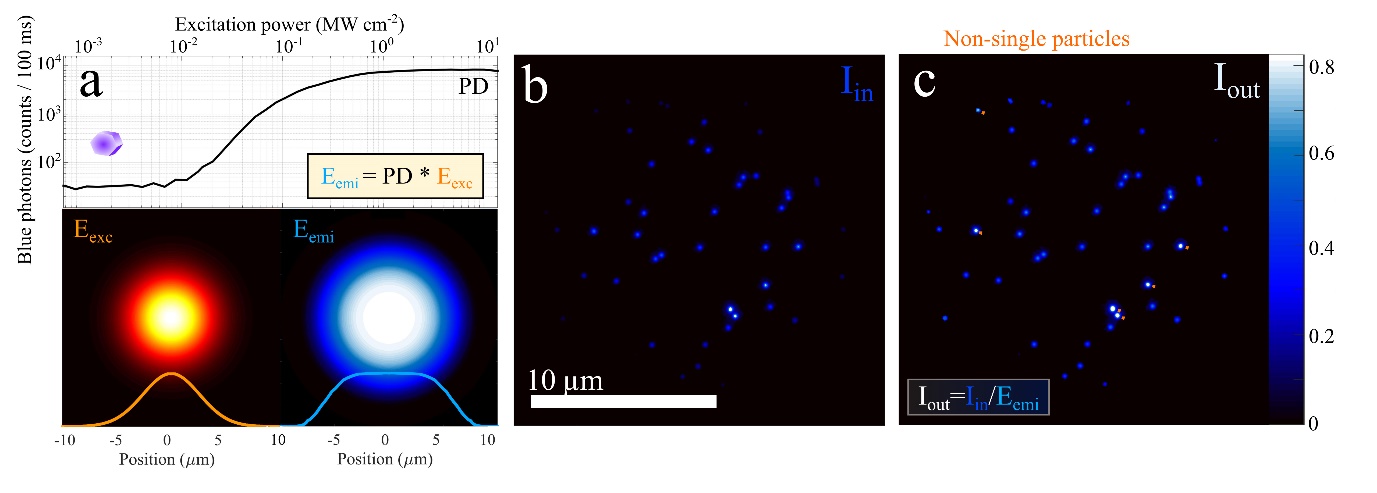


**Figure S5.** The schematic diagram and example of single UCNP determination process. (a) Excitation power dependent curve of single 4% mol Tm^3+^ doped UCNP. The bottom red spot is the excitation laser pattern. The bottom blue pattern is the emission intensity of a single UCNP in different positions of excitation beam. (b) CCD record image after LUT correction. (c) Intensity compensated image is able to be used in single UCNP determination.


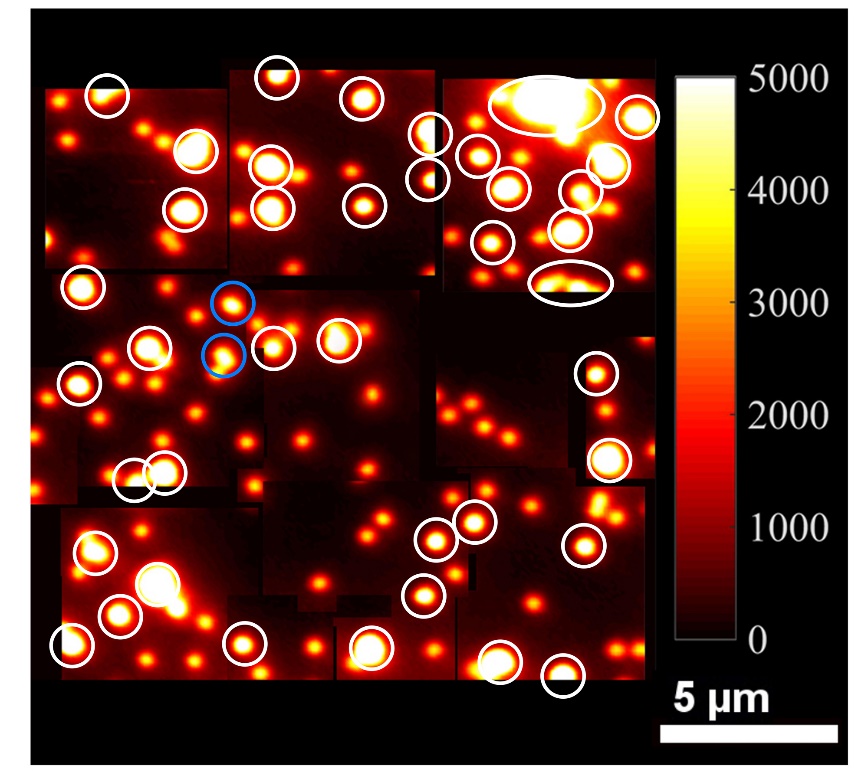


**Figure S6.** Scanning confocal result for 1% mol Tm^3+^ doped UCNP. The circles are used to label non-single particle spots calculated by confocal result. The spots labelled by blue circle can be distinguished as several single UCNPs by wide-field fluorescence microscopy.

To confirm the single UCNP determination result, we conducted scanning confocal imaging. Figure S6 show the confocal imaging of area shown in Figure 2a, where the non-single spots determined by confocal scanning are labelled by circle. All the non-single spots match with the single particle determination result shown in Figure 2a, except two spots within blue circles. The spots under blue circle cannot be distinguished due to limited resolution of confocal system by long wavelength (976.5 nm) excitation. While using wide-field fluorescence microscopy, these spots can be distinguished as several single UCNPs, since shorter emission wavelength (455 nm) provide better image resolution.

# **Section 6: Particle dynamics analysis**

**6.1 Pearson’s correlation coefficient**

To measure the interaction between the spots we calculated the Pearson’s correlation coefficient with the MATLAB function corrcoef which calculates the coefficients as according to:

$\rho(A,B)=\frac{1}{n-1}\sum_{i=1}^{n} \left(\frac{A_{i}-\mu_{A}}{\sigma_{A}}\right)\left(\frac{B_{i}-\mu_{B}}{\sigma_{B}}\right)$ (S4)

where Ai and Bi represent the (x, y) positions of the two spots A and B, respectively. μ and σ are the mean and standard deviation of the x and y positions over the entire trajectories, and n represents the number time points. The Pearson’s correlation coefficient of two particles shown in Figure 3e was calculated to be 0.46 (p < 0.05), which considered as a moderate positive correlation indicating for interaction and association between the two UCNPs, while the two UCNPs are not bounded together and keep a degree of freedom.

**6.2 MSD calculation**

To study diffusive dynamics of UCNPs within living cells, we calculated the diffusion coefficients using the mean-square displacement (MSD):

$\left\langle r^{2}(t)\right\rangle=2d\cdot D\cdot t^{\alpha}$ (S5)

where *r²* is the MSD, r is the mean distance from the starting point that a particles diffuse in time *t*, *d* is the dimensions (*d* = 2 for two-dimensional diffusion, *d* = 3 for three- dimensional diffusion), *D* is the diffusion coefficient, and *t* is the time step^5^, the diffusive exponent *α* is a parameter differentiating anomalous from normal diffusion (i.e: When *α* < 1 the process is subdiffusive and when *α*> 1 it is superdiffusive^5^). To retrieve the value of alpha the first 10% the MSD points were plotted as log (*r²*) vs log (*t*) and fitted by a straight line. The slope of the MSD determines defines the diffusive exponent *α*.

**6.3 Viscosity calculation**

The viscosity is calculated according to Stokes-Einstein equation:

$D=\frac{kT}{6\pi\eta R}$ (S6)

where *D* is diffusion coefficient, *k* is the Boltzmann constant, *T* the temperature, *R* the radius of the particle and *η* the viscosity of the medium. *R* in Figure 2 and Figure 3 is 73 nm, measured by dynamic light scattering analysis (Zetasizer Nano, Malvern, U.K.).

**6.4 Image Processing**

AVI files were converted from RGB format and only the blue channel was saved as to 8-bit Tiff files by using the FIJI software. Detection of nanoparticles and tracking algorithm analysis were executed using Imaris software v8.5 (Bitplane). Intensity threshold was applied to find the spots in each frame over time and the Cartesian coordinates were associated with frame-to-frame and linked to generate the trajectories. The MSD was calculated using the msdanalyzer MATLAB code that was written by Jean-Yves Tinevez^6^.

# **Section 7: Phototoxicity of 976.5 nm laser**

**7.1 Focused beam test**

To test the phototoxicity of 976.5 nm laser with confocal system. We focused the laser into central area of a living cancer cell through high NA (1.4) objective lens. As shown in Figure S7, when the excitation intensity higher than 27 MW cm^-2^, after 15 min of exposure the cell shows major change.

**
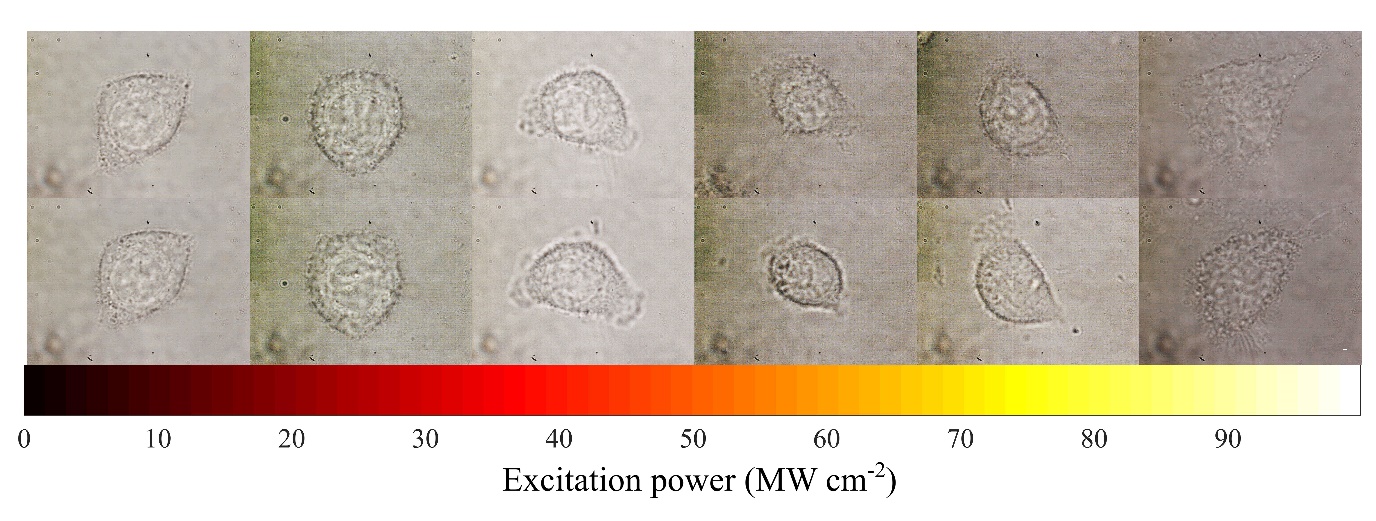
**

**Figure S7.** Phototoxicity test for focused 976.5 nm laser with power density range of 0-100 MW cm^-2^.

*Expanded beam test:*

In this paper, all the tracking is achieved under illumination of defocused beam with 5.2 µm spot size, and average power density: 0.6851 MW cm^-2^. The cell condition test under this illumination condition is shown in Figure S8, where the cell have no major change for at least 77 min, which is the longest time we can measure for living cell due to the lack of microscopy compatible incubator.


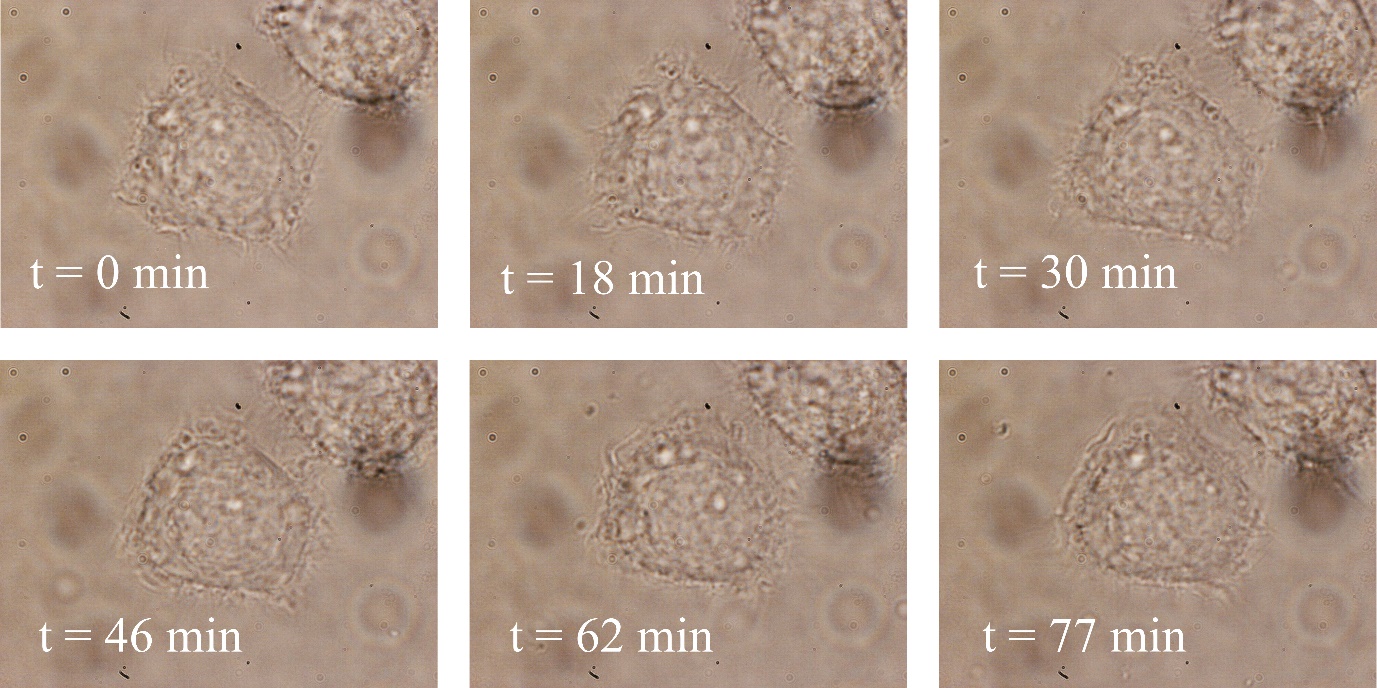


**Figure S8.** Phototoxicity test for defocused 976.5 nm laser with and average power density: 0.6851 MW cm^-2^

# **Section 8: Supplementary Movies**

**Supplementary Movie S1**

3-D single particle tracking (SPT) of USNP in dark (no bright field).

Exposure time: 150 ms; Camera gain: 4; Frame rate: 4.75; Pumping power: 400 mW;

Position start: 0 µm; Position slides number: 14; Increasing step size: 0.5 µm; height stay time: 200 ms.

**Supplementary Movie S2**

2-D single particle tracking (SPT) of USNP in dark (no bright field).

Exposure time: 150 ms; Camera gain: 4; Frame rate: 4.75; Pumping power: 400 mW;

**Supplementary Movie S3**

2-D and 3-D single particle tracking (SPT) of USNP under bright field.

Exposure time: 150 ms; Camera gain: 8; Frame rate: 4.75; Pumping power: 400 mW;

# **References**

1. Liu D, Xu X, Du Y, Qin X, Zhang Y, Ma C, et al. Three-dimensional controlled growth of monodisperse sub-50 nm heterogeneous nanocrystals. *Nat commun* 2016, **7**: 10254.

2. Liu Y, Lu Y, Yang X, Zheng X, Wen S, Wang F, et al. Amplified stimulated emission in upconversion nanoparticles for super-resolution nanoscopy. *Nature* 2017, **543**(7644): 229-233.

3. Ma C, Xu X, Wang F, Zhou Z, Liu D, Zhao J, et al. Optimal Sensitizer Concentration in Single Upconversion Nanocrystals. *Nano Lett* 2017, **17**(5): 2858-2864.

4. Clark RN. *Visual Astronomy of the Deep Sky*. Cambridge University Press, 1990.

5. Saxton MJ, Jacobson K. SINGLE-PARTICLE TRACKING:Applications to Membrane Dynamics. *Annu Rev Biophys Biomol Struct* 1997, **26**(1): 373-399.

6. Tarantino N, Tinevez J-Y, Crowell EF, Boisson B, Henriques R, Mhlanga M, et al. TNF and IL-1 exhibit distinct ubiquitin requirements for inducing NEMO–IKK supramolecular structures. *J Cell Biol* 2014, **204**(2): 231-245.
